# How shoulder immobilization after surgery influences daily activity — a prospective pedometer-based study

**DOI:** 10.1007/s00264-023-06033-z

**Published:** 2023-11-11

**Authors:** Carolin Rickert, Stefanie Ahlich, Georg Gosheger, Tobias Kalisch, Dennis Liem, Kristian Nikolaus Schneider, Sebastian Klingebiel

**Affiliations:** 1 Department of Orthopedics and Tumororthopedics, University Hospital Muenster, Albert-Schweitzer-Campus 1 BuildingA1, Muenster, Germany; 2 Sportopaedicum Berlin, Berlin, Germany

**Keywords:** Shoulder immobilization, Activity of daily living, Physical activity, Rotator cuff, Shoulder arthroplasty, Rehabilitation

## Abstract

**Purpose:**

Immobilization, especially of the lower extremity, after orthopaedic surgery has been associated with reduced physical activity. Previous interventions from our study group showed even in young, healthy people reduced activity levels after immobilization of the shoulder. Therefore, this study investigates the change in physical activity due to shoulder immobilization after a reconstructive surgery.

**Methods:**

This prospective study includes 40 patients undergoing surgery from May 2019 to December 2020. Daily activity was measured before surgery, after discharge and three weeks postoperatively each time for six days. Activity including step counts and active time were measured by Fitbit™ inspire. Range of motion before and after surgery as well as Pain (VAS) were documented.

**Results:**

Steps became significantly less immediately postoperatively with an immobilized shoulder joint than before surgery (9728.8 vs. 6022.6, *p* < 0.05). At follow-up, the number of steps increased again, but still showed a significantly lower number of steps (mean 8833.2) compared to preoperative. Patients preoperatively showed mostly an “active” activity pattern, whereas postoperatively a “low active” behaviour predominated. The proportion of sedentary behaviour (“basal activity” and “limited activity”) was almost three times higher postoperatively (12.5% vs. 30%).

**Conclusion:**

General physical activity is restricted during upper limb immobilization in adults. Therefore, activity-enhancing measures should be implemented in the early phase of rehabilitation after upper extremity surgery.

## Introduction

There is a linear relationship between one’s physical activity (PA) habits and health status. Physical inactivity is the fifth largest risk factor for mortality after arterial hypertension, nicotine abuse, elevated blood glucose, and obesity (1). PA and exercise are indispensable components of an independent and healthy lifestyle. Not only the positive effect of physical activity on various diseases and functional limitations has been widely documented in the literature (2). The prevention of a large number of diseases by regular physical activity has also been demonstrated with a high level of evidence (IA), and most of them are amenable to therapy by physical activity (3). These include chronic kidney disease, various cancers, peripheral occlusive disease, depression, and osteoporosis (3). PA has been shown to reduce significantly mortality and morbidity of each disease by up to 30–40% (Position paper, European Federation of Sports Medicine Associations). In addition to health-promoting effects on physical condition, physical activity also has a positive effect on a person’s psychological condition. For example, an increase in physical activity in people diagnosed with depression shows a positive effect on the expression of symptoms. In addition to influencing symptom expression, physical activity additionally reduces the risk of disease recurrence (4).

Additionally to the importance of physical everyday activity for prevention and health promotion, PA is also an essential component in orthopaedic rehabilitation. Especially in this field, secondary prevention is gaining great importance, as patients’ inactivity may be particularly distinct during this period, for example due to pain or even immobilization of the operated limb. It is not only the physiotherapeutic or other therapeutic measures that are of great importance to reintegrate the patient into professional, sporting and other everyday life, but also the lifestyle in everyday life for a faster recovery as well as secondary prevention during rehabilitation is essential (11). Patients with physical limitations in particular tend to be less active than the general population, making a physically active lifestyle even more important for these individuals (12,13).

Orthoses are regularly prescribed to immobilize the operated limb as part of conservative therapy, but also after surgical interventions in orthopaedics. Studies show that a lower level of physical activity and a higher proportion of sedentary behaviour can be observed in people who sustain an orthopaedic injury up to 18 months after the injury [1, 2].

With regard to immobilization of the upper limb, there is insufficient research on the effects on physical activity. Maggio et al. were able to measure significantly lower physical activity in 35 children with upper limb plaster supply compared to a healthy control group, so that upper limb immobilization in children led to a reduction in physical activity with a subsequent decrease in energy expenditure (121). A preliminary pilot study from our study group showed even in young, healthy volunteers immobilization of the shoulder in an orthosis for two days led to significantly reduced activity levels [3].

Further studies are lacking in the literature. Therefore, the aim of this study was to investigate the physical activity of adults after shoulder surgery and to evaluate the validity and practicability of the activity monitors used.

## Materials and methods

### Participant recruitment and study design

Forty consecutive patients (20 women, 20 men) suffering from rotator cuff tear, omarthrosis or defectarthropathy have been included in this prospective study from outpatient clinic at a university hospital. Mean age was 61 years (42–78 years). The local Ethics Committee (2019–554-f-S) approved (the study protocol adheres to the ethics guidelines of the Declaration of Helsinki). The study was registered in DRKS (DRKS00017636). Contents were explained both orally and in written form and all participants gave their written informed consent.

Inclusion criteria were diagnosis of rotator cuff tear, omarthrosis or irreparable cuff tear. Exclusion criteria was walking impediment.

### Surgery and immobilization of the shoulder joint

In all patients, surgery was performed in beach-chair position under general anaesthesia. Depending on diagnosis, an arthroscopic reconstruction of rotator cuff or reverse shoulder arthroplasty was made. Discharge from the hospital took place after two days (5 days in case of arthroplasty).

**Fig. 1:**
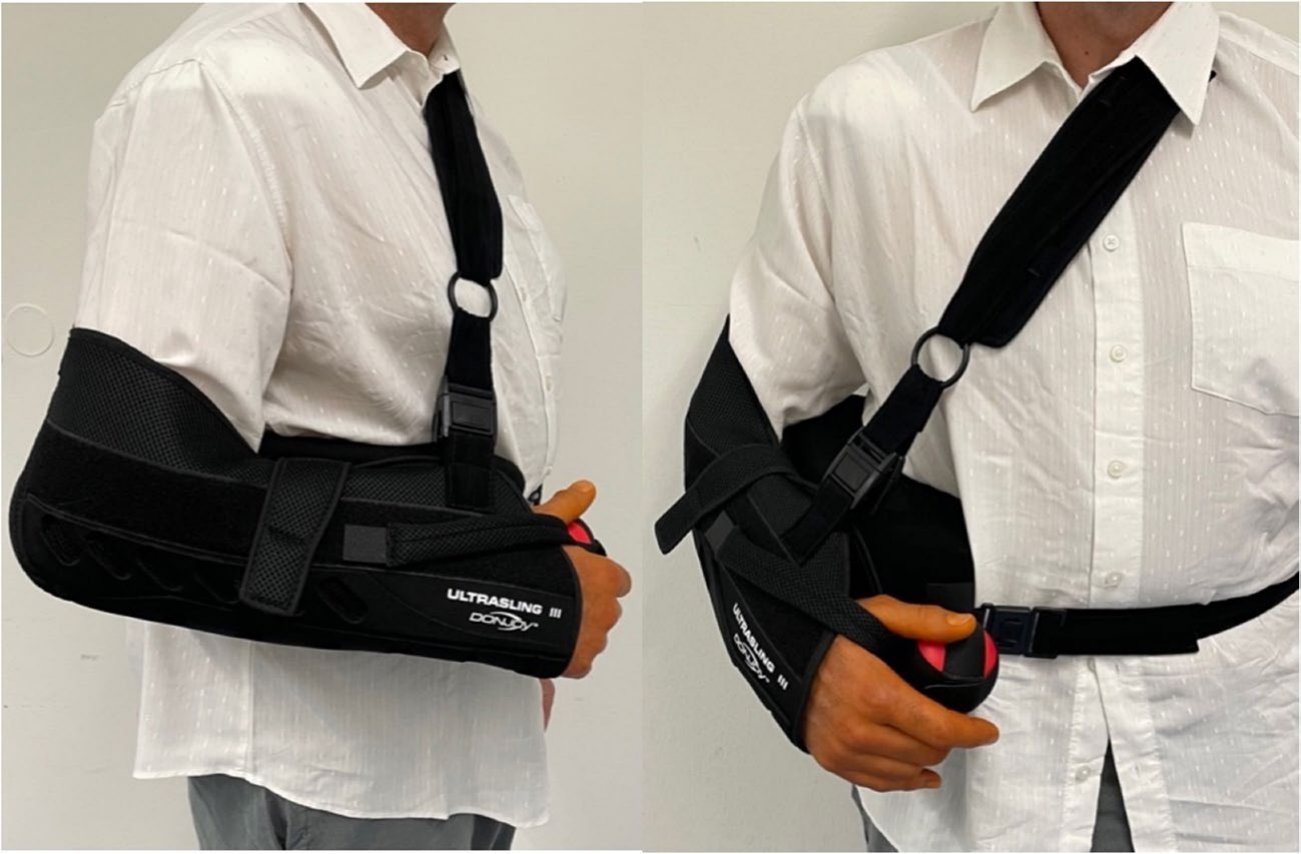
Donjoy Ultrasling III shoulder joint orthosis

After surgery the shoulder joint was immobilized by the DONJOY GLOBAL shoulder joint orthosis (DJO Ultrasling III) (see Fig. 1) for four to six weeks with an abduction of 10–15° in the lower arm sling with a connected body pillow. Physiotherapy was carried out along a standardized rehabilitation scheme for shoulder mobilization.

### Objective measurement using wearable fitness tracker

For objective measurement, Fitbit™ (Inspire, United States, San Francisco, CA, USA) was used. It is a wrist-worn accelerometer measuring step counts, active time and calories burned. Participants were instructed by the physician in use of the device. They were supposed to wear their assigned Fitbit™ device all day, removing it only for body care or charge the device. The participants had worn the Fitbit™ for six consecutive days on the non-immobilized arm. This period was determined based on recommendations that at least 1 day from a weekend should be included for gathering accelerometer data recorded by employed persons. Regarding the length of data gathering periods, a minimum of four days is considered [4, 5]. Activity measurement was carried out with the Fitbit™ at three different measurement times on six consecutive days each.

The first measurement took place on six consecutive days directly after the indication for surgery. After information and a written declaration of consent, the patients received an accelerometer personalized to their physical parameters (height, weight, date of birth, gender and age).

The second measurement followed the day after discharge from hospital. At this time, the operated arm was immobilized in the orthosis (DONJOY® GOBAL, DJO Ultrasling III).

At the beginning of the third postoperative week, the third measurement was carried out, also with immobilized shoulder, in order to exclude any influence of immediate postoperative restrictions (pain, side effect of anaesthesia).

Daily number of steps and activity in active minutes were documented. Fitbit™ device calculates active minutes using METs (metabolic equivalents) based on the entered data regarding body weight, height and gender. Active minutes were recorded as at least ten min of continuous moderate to vigorous activity.

Activity was documented according to the classification system of Tudor-Locke (see Table 1).

**Table 1:** Classification of activity according to Tudor-Locke et al.

Intensity	Designation	Steps in healthy adults
Sedentary	Basal activity Limited activity	≤ 2499 2500–4999
Light	Low active Somewhat active	5000–7499 7500–9999
Moderate	Active	10,000–12,499
Vigorous	Highly active	≥ 12,500

For the first 20 patients included, push notifications regarding exercise progress were turned on in the Fitbit™ device settings; for the other 20 patients, these push notifications were turned off.

Objective measurement of range of motion pre- and postoperatively as well as pain by visual analog scale (VAS) were documented.

### Statistical analysis

For statistical analysis, Microsoft Office 2016 (Microsoft Corporation, Redmond, WA, USA), Microsoft Excel 2016 (Microsoft Corporation, Redmond, WA, USA) and SPSS 23 for Mac (IBM Corporation, NY, USA) were used.

After checking the application requirements, analysis of variance (ANOVA) was performed using the following analytical test procedures:

- Friedmann test for non-parametric data.

- Wilcoxon test for connected samples.

## Results

Forty consecutive patients (20 male, 20 female) were included in this prospective study. The mean age was 61 years (± 8.1). Twenty-seven patients underwent rotator cuff repair and thirteen patients received shoulder arthroplasty (see Table 2).

**Table 2:** Demographic data of study population

	All	Female	Male
N	40	20	20
Age (years)	61 (± 8.1)	63 (± 7.9)	58 (± 7.7)
BMI (kg/m^2^)	30 (± 7.9)	31 (± 7.8)	29,1 (± 7.9)
Arthroplasty	13	10	3
RC Repair	27	10	17

In our study group, there was a significant decrease in pain and physical activity regardless of the surgical procedure (Table 3).

**Table 3:** Results of pain (VAS), steps and active minutes in all patients

	Median (95% CI)	*P* value
Pain preOP	7 (6.32/7.13)	
Pain FU	1 (0.84/1.36)	< 0.05
Steps preOP	9729 (8261/10528)	
Steps postOP	6023 (5491/7400)	< 0.05
Steps FU	8833 (8114/10270)	< 0.05
Activity preOP	25.2 (20.7/34,3)	
Activity postOP	17.6 (17.5/32.3)	< 0.05
Activity FU	24.35 (23.8/42.3)	n.s

As expected, range of motion of the shoulder joint was limited in all directions after surgery due to immobilization (see Table 4).

**Table 4:** Range of motion (degree) of all patients before and after surgery

	Abd preOP	Flex preOP	ARO preOP	IRO preOP	Abd postOP	Flex postOP	ARO postOP	IRO postOP
Mean	86	126	55	53	94	114	41	50
STD	33.83	46.67	22.38	24.36	22.11	31.77	21.92	17.78
Max	180	180	90	90	180	180	80	80
Min	30	40	10	0	70	60	0	20

**Fig. 2:**
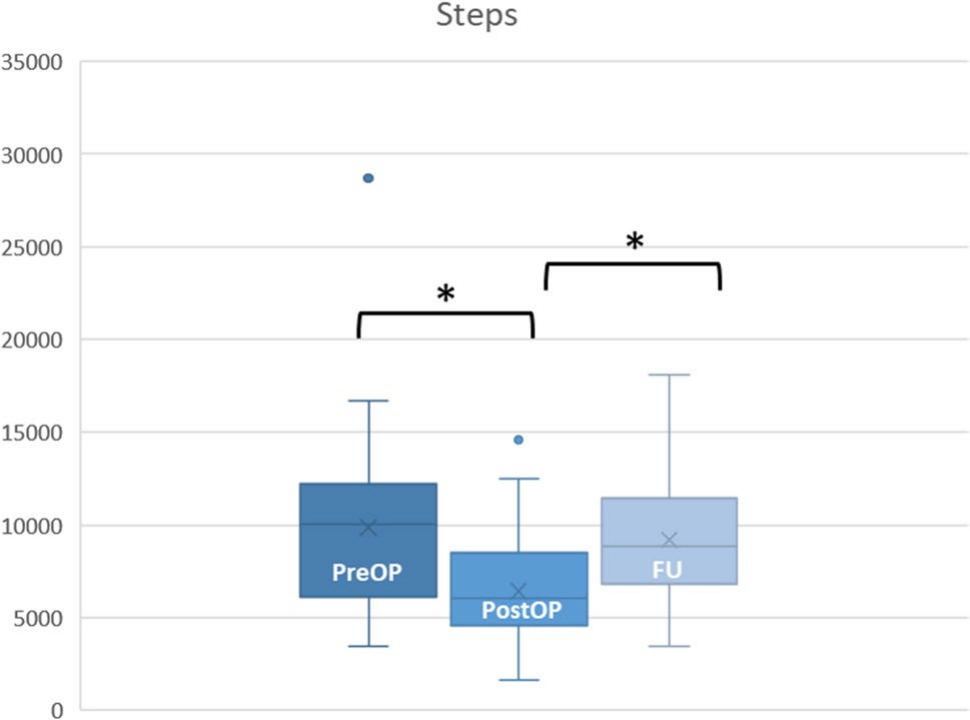
Boxplot representation of mean values of steps at measurement points (*: significant)

Patients took significantly fewer steps immediately postoperatively with immobilized shoulder joint than before surgery (9728.8 vs. 6022.6, *p* < 0.05). At FU three weeks postoperatively, the number of steps increased again, but still showed a significantly lower number of steps with a mean of 8833.2 steps compared to the measurement before surgery (see also Fig. 2). After correction for the factors BMI and age, there was no correlation between the increase in number of steps and pain reduction.

Overall, lifestyle thus changed according to the categories of Tudor-Locke: regarding the mean values, an average *somewhat active* behavior was shown preoperatively. The activity then decreased postoperatively and fell into *low active* category. At follow-up, average activity rose again to *somewhat active* initial level. A *somewhat active* as well as *low active* behaviour pattern corresponds to a light intensity of physical activity.

After assessing the absolute and relative frequencies of the individual categories, it became apparent that patients with an *active* activity pattern made up the largest proportion preoperatively, while a *low active* behavior predominated postoperatively. At follow-up, the majority of patients were *somewhat active*. The proportion of sedentary behavior (basal activity and limited activity) was significantly lower in preOP with 12.5% versus 30% postOP. At FU, this behavior decreased to 10%. (See Table 5). Only postOP four patients showed basal activity with less than 2499 steps. None of the patients took place in this lowest activity level before surgery and at follow-up.

**Table 5:** Absolute and relative frequency distribution of the activity levels according to Tudor-Locke at all measurement points

Classification by Tudor-Locke	Pre OP	Post OP	Follow-up
Basal activity	0 (= 0%)	4 (= 10%)	0 (= 0%)
Limited activity	5 (= 12.5%)	8 (= 20%)	4 (= 10%)
Low active	9 (= 22.5%)	17 (= 42.5%)	8 (= 20%)
Somewhat active	6 (= 15%)	7 (= 17.5%)	14 (= 35%)
Active	11 (= 27.5%)	3 (= 7.5%)	7 (= 17.5%)
Highly active	9 (= 22.5%)	1 (= 2.5%)	7 (= 17.5%)

**Fig. 3:**
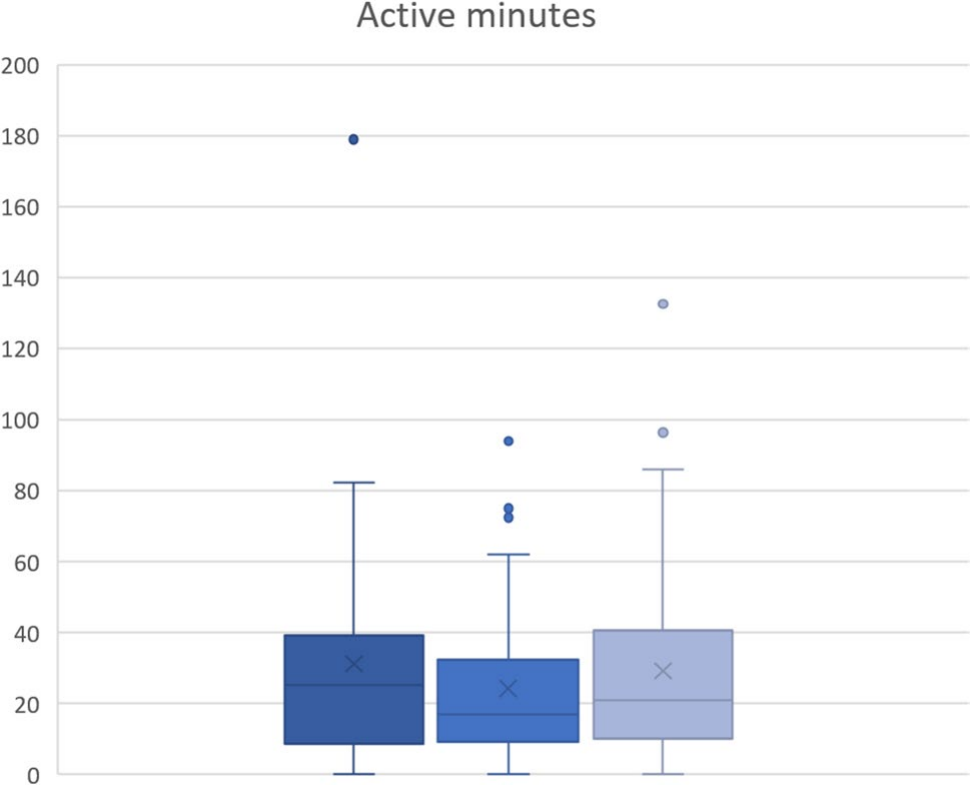
Boxplot representation of mean values of active minutes

In addition to the steps, the active minutes were also evaluated. There was less activity directly postoperatively with an average of 17.6 min compared to preoperatively (25.2 min) (Fig. 3). At FU, an increase in active minutes to almost the preoperative level with an average of 24.3 active minutes was documented. Overall, however, the results were not significant (*p* = 0.713).

## Discussion

This study confirmed the hypothesis that patients show less activity due to shoulder surgery, which was evaluated by the number of steps per day. Patients took significantly fewer steps immediately postoperatively with an immobilized shoulder joint than before surgery (9728 ± 3543 vs. 6022 ± 2986) (*p* < 0.05). During the follow-up period, physical activity continued to be limited by the shoulder orthosis and showed a significantly lower number of steps with a mean of 8833 ± 3371 steps compared to the measurement before surgery. The *somewhat active* lifestyle, which had already existed preoperatively, thus initially deteriorated to a *less active lifestyle*. The proportion of sedentary behaviour (*basal activity* and *limited activity*) was almost three times higher postoperatively (12.5% vs. 30%).

In the literature, most of the studies based on limited activity after surgery of the lower extremity and only a few on the upper extremity. For example, Beckenkamp et al. initially observed an increase in physical activity in patients after an ankle fracture in the first month after the end of immobilization of the affected ankle. However, in the follow-up period up to the sixth month, activity remained below the level of physical activity recommended by the WHO [6]. Similar results were obtained by Webber et al. who used an accelerometer to examine the sedentary behaviour and physical activity of patients both preoperatively and 1 year after implantation of a knee joint prosthesis [7]. The comparison group consisted of patients with gonarthrosis who had not yet received endoprosthetic treatment. There was no significant difference in the preoperative and postoperative extent of sedentary behavior. In comparison, this behaviour was consistent with the patients without knee joint prosthesis. Only a marginal increase in physical activity could be documented in the group of operated participants, which, however, did not correspond to the amount of exercise of healthy adults of the same age. Luna et al. [8] also showed an early, postoperative reduction in physical activity in patients after implantation of a total hip or knee joint endoprosthesis. In this prospective study of 40 patients, the authors were able to show that physical activity was significantly reduced two and three weeks after surgery compared to the preoperative state. Slight postoperative improvements were observed in high-intensity activities, but with a large interindividual variation. Approximately 30% of HTEP patients and 20% of TKA patients showed reduced and decreasing activity throughout the study period. No single major factor for reduced postoperative physical activity was identified, although anemia, obesity, inflammatory stress response and postoperative pain were associated with poor recovery.

In our preliminary pilot study, wearing of a shoulder orthosis already affected the daily physical activity of 20 healthy young volunteers by significantly lowering the step cycles (*p* = 0.000). In this investigation, wearing a shoulder orthosis significantly (*p* = 0.000) contributed to the activity level decreasing from *moderate* activity to the harmful level of *light* activity [3].

In our present study, there was no increased motivation in terms of daily activity from the Fitbit™ device worn. The literature shows mixed results of the effect of wearable fitness tracker (WFT) on motivation to move more. For example, in a study of overweight adolescents, WFT use was associated with increased self-determined motivation for PA and increased muscular endurance and strength [9]. Conversely, adolescents who wore a Fitbit™Charge device for 8 weeks reported lower autonomous motivation for PA [10]. While the social aspect of the Fitbit™ mobile app (e.g., adding friends and participating in contests) increased feelings of connectedness, participation in contests per se did not increase forms of controlled motivation [10]. In contrast, in a study of adults, adding a WFT positively influenced motivation for PA [11]. Another study showed that Fitbit™users reported an increase in motivation for PA compared to a control group over a 13-month period [12]. These studies thus suggest that the use of WFTs may improve motivation for PA in adults, older adults and overweight adolescents, although further research is needed to investigate this issue further.

To date, there are no studies in the literature documenting physical activity after upper limb immobilization. Only Maggio et al., in a longitudinal matched case–control study of 35 children and adolescents with an upper limb fracture and healthy matched controls (with no history of fracture) aged ten to 16 years, showed that subjects with upper limb fractures achieved significantly lower PA scores and spent more time in sedentary activities. Total activity was significantly lower (− 30.1%) in the children with upper limb fractures compared to the healthy controls [13].

Some studies showed that FitBit® devices provided comparatively accurate estimates of sedentary activity compared to validated accelerometers, but overestimated moderate to vigorous activity in free-living conditions [14]. In addition, FitBit® test-rest reliability was found to be dependent on the type of activity and to have greater variation between measurements compared with other wearable devices [15]. Despite these limitations, the FitBit® is suitable for the intended purpose of our study considering its advantages in terms of compliance, handling, and data collection.

In the present investigation, objective measurement of step activity in patients who underwent shoulder surgery was well feasible using a Fitbit™ device. No technical problems occurred with regard to data processing. Furthermore, no restrictions were reported due to wearing the devices, so that the monitors did not affect the patients’ daily activities.

## Limitations

Due to the small sample (*N* = 40), conclusions cannot be generalized. Further studies are necessary to be able to draw conclusions about the general population. There is no comparability with other studies since there are no comparable studies in the literature.

## Conclusion

Our results show restricted general physical activity during upper limb immobilization in adults. Despite free mobility and mobilization possibilities of the lower extremity, patients in the early postoperative interval were less active and showed an increased sedentary behavior, so that immobilization of the shoulder joint has an influence on the activity level. Postoperative rehabilitation is a good way to promote physical activity because therapy focuses on regaining mobility and often includes structured exercise programs or sports activities. Thus, it might be a good strategy to incorporate such activities into daily life at this time to continue to maintain them after rehabilitation and to internalize a more active lifestyle. Therefore, activity-enhancing measures should be implemented in the early phase of rehabilitation after surgery of upper extremity.

## Data Availability

The data that support the findings of this study are available on request from the corresponding author, [CR]. The data are not publicly available due to containing information that could compromise the privacy of research participants.
